# Dissipation Kinetics and Risk Assessment of Spirodiclofen and Tebufenpyrad in *Aster scaber* Thunb

**DOI:** 10.3390/foods12020242

**Published:** 2023-01-05

**Authors:** Ramesh Kumar Saini, Yongho Shin, Rakdo Ko, Jinchan Kim, Kwanghun Lee, Dai An, Hee-Ra Chang, Ji-Ho Lee

**Affiliations:** 1 Department of Crop Sciences, Konkuk University, Seoul 05029, Republic of Korea; 2 Department of Applied Biology, Dong-A University, Busan 49315, Republic of Korea; 3 Bio Division, Korea Conformity Laboratories, Incheon 21999, Republic of Korea; 4 Department of Food & Pharmaceutical Engineering, Graduate School of Hoseo University, Asan 31499, Republic of Korea

**Keywords:** pre-harvest residue limits (PHRLs), hazard quotient, *Doellingeria scabra* (Thunb.) Nees, pesticide, maximum residue limit, multiple reaction monitoring (MRM)

## Abstract

The dissipation kinetics of spirodiclofen and tebufenpyrad after their application on *Aster scaber* Thunb were studied for 10 days, including the pre-harvest intervals. Spirodiclofen and tebufenpyrad were used in two greenhouses in Taean-gun, Chungcheongnam province (Field 1) and Gwangyang-si, Jeollanam province (Field 2), Republic of Korea. Samples were taken at 0, 1, 3, 5, 7, and 10 days after pesticide application. The method validations were performed utilizing liquid chromatography (LC)-tandem mass spectrometry (MS/MS). The recoveries of the studied pesticides ranged from 82.0–115.9%. The biological half-lives of spirodiclofen and tebufenpyrad were 4.4 and 3.8 days in Field 1, and 4.5 and 4.2 days in Field 2, respectively. The pre-harvest residue limits (PHRLs; 10 days before harvesting) of *Aster scaber* were 37.6 mg/kg (Field 1) and 41.2 mg/kg (Field 2) for spirodiclofen, whereas the PHRLs were 7.2 (Field 1) and 3.6 (Field 2) for tebufenpyrad. The hazard quotient for both pesticides at pre-harvest intervals was less than 100% except in the case of spirodiclofen (0 day).

## 1. Introduction

Pesticides are widely used to control pests on crops effectively. The use of pesticides has resulted in increased productivity of cultivated crops. Thus, pesticides have become an economically essential agricultural resource for modern agriculture [1,2,3]. Despite the necessity for pesticides, the risks related to pesticide use and their residues in humans and the environment are being reported [4,5]. Therefore, considering the risks to consumers, the environment, and the productivity of crops, agricultural chemical management safety policies are being implemented nationwide. Pesticide residues in domestically distributed and imported agri-foods are being monitored in an effort to decrease the marketing and consumption of crops that exceed the maximum residue limit (MRL). Moreover, the Ministry of Food and Drug Safety has established and applied the pre-harvest residue limit (PHRL) in the production stage to systematize the management of pesticide residues before shipment and on a broader scale, to minimize damage to producers and consumers due to the distribution of substandard crops. According to the recommendations on the safe use of pesticides, PHRL is calculated and established using the biological half-life and decay constant, which are calculated each day before shipment after spraying the chemical [6]. Therefore, to provide safe food to consumers by preventing substandard crops from exceeding pesticide residue limits at the domestic distribution stage and to produce agricultural products meeting the pesticide residue tolerance limit of the exporting country, the establishment and management of PHRL is crucial [6].

Spirodiclofen, 3-(2,4-dichlorophenyl)-2-oxo-1-oxaspiro [4.5]dec-3-en-4-yl 2,2-dimethylbutyrate (Table A1) is an insecticide belonging to the chemical class of ketoenols or tetronic acids [7]. Spirodiclofen inhibits lipid biosynthesis and is approved for use on citrus, grapes, pears, nuts, and other plants in many countries to control pests and mites [7,8]. Tebufenpyrad is an electron transport chain inhibitor and a pyrazole acaricide that effectively inhibits *Tetranychus*, *Panonychus*, *Origonychus*, and *Eotetranychus* species [9].

*Aster scaber* Thunb. (Syn. *Doellingeria scabra* (Thunb.) Nees) is a perennial herb of the Asteraceae family, widely cultivated in the temperate region of Korea for culinary uses. It is a rich source of vitamins, minerals, and essential amino acids, which help minimize the incidence of chronic diseases [10]. It also contains a large amount of unsaturated fatty acids, such as linolenic acid, which lowers blood cholesterol [11].

Dissipation kinetics is used to determine the in-plant biological half-lives and pre-harvest intervals (PHIs). Therefore, in this study, the basic data for establishing the pesticide residue tolerance standards in the production stage were obtained by measuring the residual amount each day during cultivation after spraying *Aster scaber* with spirodiclofen and tebufenpyrad, which are used to eliminate *Tetranychus urticae* Koch and *Tetranychus kanzawai*. Through a risk assessment during the PHI period, the study sought to identify the risks of ingesting *Aster scaber* foods sprayed with spirodiclofen and tebufenpyrad.

## 2. Materials and Methods

### 2.1. Test Chemicals and Reagents

The pesticide standards, spirodiclofen and tebufenpyrad, were purchased from Kemidas, Gunpo-si, Gyeonggi-do, Republic of Korea. The chemicals spirodiclofen (36% wettable powder, Bayer CropScience, Seoul, Republic of Korea) and tebufenpyrad (10% emulsifiable concentrate, Syngenta, Seoul, Republic of Korea) were purchased as commercial products. The chemical structures and physicochemical properties of the two pesticides are shown in Table A1 [12]. An HPLC grade solvent was purchased from J.T. Baker^®^ (Avantor Performance Materials Korea Ltd., Suwon-Si, Republic of Korea). The solid reagent formic acid was purchased from Merck Ltd., Seoul, Republic of Korea. The QuEChERS extraction kit (MgSO_4_ 4 g, NaCl 1 g) was purchased from Chiral Technology Korea, Daejeon, Republic of Korea.

### 2.2. Field Trial

The field trials were carried out from April 2019 to June 2019, and the same cultivar of *Aster scaber* (Asia Seed Co., Ltd., Seoul, Republic of Korea) was used for both fields. Accounting for geographical differences, facility cultivation sites distanced more than 20 km apart (latitudinally) were selected as field trial sites with the locations in Taean-gun, Chungcheongnam province (Field 1), and Gwangyang-si, Jeollanam province (Field 2). Seeding was carried out on 20 June (Field 1) and 28 April (Field 2). The experimental plot was set at 10 m^2^ per repetition and consisted of 3 treatment plots and one non-treatment plot. A small engine knapsack-type sprayer (MSB1015Li, Maruyama, Tokyo, Japan) was utilized for chemical spraying after diluting and preparing the test chemicals in accordance with the safe use standard of agricultural chemicals (Table 1). Samples were taken 0, 1, 2, 3, 5, 7, and 10 days after pesticide application. An appropriate size for the sample was established per day, and an amount of ≥1 kg consisting of at least 12 units was collected. The collected samples were placed in a polyethylene bag, labeled with the chemical name and collection date, stored in an icebox, and immediately delivered to the lab.

### 2.3. Sample Preparation

The samples transported to the laboratory were weighed and then shredded for sample preparation. Before being homogenized with dry ice and a homogenizer, the shredded samples were kept in a refrigerator (below 20 °C) for more than 48 h. Homogenized samples were kept frozen (below 20 °C) until analysis.

### 2.4. Quality Assurance and Quality Control

The method limit of quantification was determined by instrumental analysis of a standard solution with a signal-to-noise ratio (S/N) of ≥10. The LC-MS/MS parameters utilized for the quantitative analysis of spirodiclofen and tebufenpyrad are shown in Table A2. The stock solutions (100 mg/L) of spirodiclofen and tebufenpyrad were prepared in acetonitrile. The prepared stock solution was matrix-matched with *Aster scaber* extract at a ratio of 1:1 to prepare matrix-matched standard solutions of 0.025, 0.05, 0.1, 0.5, 1, and 5.0 mg/L. The calibration curve was drawn by plotting the peak area against the solution concentrations, and the linearity was determined using the regression equation and the coefficient of determination (r^2^).

For both pesticides, the recovery rate test was repeated thrice at concentrations of 0.01 and 0.1 mg/kg. An amount of 10 g of the sample was treated with the standard solution to achieve these concentrations, followed by vigorous shaking for 30 min with 10 mL of acetonitrile. The extract was added to the QuEChERS extraction kit (MgSO_4_ 4 g, NaCl 1 g), shaken, and centrifuged at 4000× *g* for 10 min. Then, 1 mL of the supernatant was carefully taken out, mixed with 1 mL of acetonitrile, transferred to a 2 mL autosampler vial, and utilized for the LC-MS/MS analysis (Table A2).

### 2.5. Storage Stability and Residual Amount per Date

Storage stability was conducted in 3 repetitions by adding 0.1 mg/kg of spirodiclofen and tebufenpyrad standard solutions to untreated samples of 10 g each, uniformly mixing, freezing (below 20 °C), and testing the recovery rates after 162 days and 150 days, respectively. The samples were tested using the same method for the sample analysis that was used in the recovery rate test. The sample analysis used the same preparation and instrumental analysis as the recovery rate test to examine the daily residual amounts of spirodiclofen and tebufenpyrad during the *Aster scaber* cultivation period.

### 2.6. Calculation of Biological Half-Life and Tolerance Limit of Residue at the Production Stage

The residual amount reduction constant and biological half-lives of spirodiclofen and tebufenpyrad of *Aster scaber* were calculated via regression analysis of their daily residual amounts. After confirming the significance of the regression equation and reduction constant through the *F*-test and the *t*-test, the lower limit of the reduction constant was calculated at the 95% confidence interval. The regression analysis was performed according to the guidelines of the Ministry of Food and Drug Safety, Republic of Korea [13]. The PHRL at the production stage was calculated by estimating the daily residual amount up to 10 days before shipment based on the residual tolerance standards for spirodiclofen and tebufenpyrad of *Aster scaber*.

### 2.7. Risk Assessment

Risk assessments were conducted on the spirodiclofen and tebufenpyrad residues in *Aster scaber*. The estimated daily intake (EDI) was calculated by multiplying the food consumption and initial pesticide concentration of *Aster scaber* on days 0 and 7, and dividing it by the average body weight [14]. The hazard quotient (HQ) value was calculated using the acceptable daily intake (ADI) and was used for the risk assessment.

## 3. Results and Discussion

### 3.1. Temperature, Humidity, and Growth Characteristics within the Facility during Aster scaber Cultivation

During the field-testing period, the mean humidity of Fields 1 and 2 were 76.4 ± 4.7% and 77.4 ± 14.1%, respectively. Similarly, the mean temperatures of Fields 1 and 2 were 16.9 ± 2.6 °C and 17.1 ± 1.8 °C, respectively. The mean weight of 20 plants of *Aster scaber* from day 0 to day 10 after pesticide spraying in Field 1 was 131.0 ± 9.2 g on day 0 and 160.3 ± 4.9 g on day 10. While in Field 2, no significant differences were observed, with 137.3 ± 6.1 g on day 0 and 141.3 ± 5.0 g on day 10. This weight difference was >5 times compared to a previous *Aster scaber* study where the weight of a single unit increased from 0.9 g (day 0) to 4.8 kg (day 10) during the test period (10 days) [15]. In our study, we found that *Aster scaber* cultivated in Fields 1 and 2 only grew to a certain size before slowing down. This is in contrast to cucumbers and broccoli, which grow continuously and rapidly [16].

### 3.2. Method Validation

The analytical limit of quantification for spirodiclofen and tebufenpyrad in *Aster scaber* was 0.01 mg/kg for both pesticides. The calibration curve of the standard solutions was conducted via regression analysis of the peak area within the concentration range (0.0025–0.5 mg/L), and the linearity within the concentration range was confirmed by the coefficient of determination (r^2^). For spirodiclofen, the regression equations calculated through regression analysis were y = 10,601.7x + 30.2 (Field 1) and y = 13,435.6x + 26.5 (Field 2), whereas for tebufenpyrad, the equations were y = 2879.5x − 1.8 (Field 1) and y = 2785.7x + 4.0 (Field 2). The calibration curve equation of spirodiclofen and tebufenpyrad was y = 10,601,678.7x + 30,166.1 and y = 2879,505.1x + 1782.6, respectively. The coefficients of determination of the calibration curves of spirodiclofen and tebufenpyrad were both ≥0.999, thereby confirming high linearity. According to the results of the recovery rate tests at concentrations of 0.1 and 1.0 mg/kg, the average recovery rates of spirodiclofen were 100.2% and 104.1%, respectively, whereas those of tebufenpyrad were 89.1% and 103.3%, respectively. The coefficients of variation (CV) were <10%, which was within the acceptable range for the persistence test’s analytical method verification criteria (70–110% recovery rate and CV within 20%) for establishing the PHRL (Table 2). During quantitative analysis by LC-MS/MS of spirodiclofen and tebufenpyrad in *Aster scaber*, no interference peaks were observed in the recovery and untreated samples.

For the storage stability test conducted at a concentration of 1.0 mg/kg, spirodiclofen showed a recovery rate of 101.8% ± 1.8% after 162 days of storage and tebufenpyrad showed a recovery rate of 104.2% ± 4.9% after 150 days of storage, confirming that there was no degradation or loss of both components during the storage period of the samples.

### 3.3. Characteristics of Pesticide Residues in Aster scaber

Several studies have reported factors affecting pesticide persistence in crops, including (1) geographical locations and weather conditions of the cultivation area, (2) function, formulation, and application method of pesticides, (3) curvature of the surface of the crop, (4) surface area to weight ratio of the crop, (5) shape and growth rate, (6) amount and shape of villi in crops, (7) composition of the wax layer on the surface of the crop, and (8) the crop cultivation methods [17,18,19,20]. In the present study, *Aster scaber* did not show a significant change in weight during the test period; thus, the possibility of the disappearance of pesticides due to the dilution effect (due to weight gain) can be eliminated.

Hong et al. [15] reported that test pesticides have a tendency to disappear during the cultivation period of *Aster scaber* as a result of the dilution effect of the treated chemical caused by the growth rate of *Aster scaber* and its reducing effect on the residual concentration of the pesticide. The dilution effect due to growth was deemed insignificant in this study, as confirmed by the slow growth of the *Aster scaber*. 

The difference in residues between Field 1 and Field 2 after treatment with spirodiclofen and tebufenpyrad during the cultivation period of *Aster scaber* was determined. Considering the difference in residue between Field 1 and Field 2, the residue amounts of Field 1 were 1.8 times and 2.9 times higher than that of Field 2 for spirodiclofen and tebufenpyrad, respectively. These results are similar to those of a previous study on *Aster scaber* conducted in the same area where the residue amount of Field 1 was approximately 1.5 times higher than that of Field 2 [16]. This difference in residue amount is considered to have occurred due to the difference in the initial residue amounts of the pesticides. The half-life per region for spirodiclofen was 4.4 and 4.5 days in Fields 1 and 2, respectively. Meanwhile for tebufenpyrad, it was 3.8 and 4.2 days, respectively, indicating no significant difference between the half-lives of the two fields, unlike the residue amounts. It is thought that the half-lives are similar because the crops used in both fields were identical, and the difference between temperature and humidity had little effect on crop growth. In conclusion, even if the initial residue amount differs, if there is no difference in the growth environment, including the crop temperature and humidity, there will be no difference in the pesticide’s half-life within the crop’s body.

The initial residue amount after spirodiclofen treatment was 14.2 and 8.9 mg/kg in Fields 1 and 2, respectively. Meanwhile for tebufenpyrad, it was 4.9 and 2.0 mg/kg, respectively. This shows that the initial residue amount of spirodiclofen was about 2.9 times higher than that of tebufenpyrad in Field 1 and 4.5 times higher in Field 2. When spraying, the safety standards for both spirodiclofen and tebufenpyrad were the same with up to two treatments, seven days before harvest. Spirodiclofen wettable powder was sprayed at a 4000-fold dilution at 36% content, whereas tebufenpyrad emulsifiable concentration was applied with a 2000-fold dilution at 10% content. Thus, the total amount of spirodiclofen’s active ingredient was about 1.8 times greater than that of tebufenpyrad (Table 1). Thus, one of the primary causes of the difference in the initial residue amount of spirodiclofen and tebufenpyrad was determined to be a difference in the total sprayed amount (TSA) of the active ingredient. To account for the difference in TSA, the normalized values (NVs) obtained by dividing the residue amount of each pesticide by TSA were applied (Table 3). The NV of spirodiclofen on day 0 was 15.8 (Field 1) and 9.9 (Field 2), whereas that of tebufenpyrad was 9.8 (Field 1) and 4.0 (Field 2), indicating that the difference in the original residue amount was reduced (concentration not accounting for the amount of active ingredient applied). Although the amount of active ingredient applied was corrected, the difference between the two fields could be due to various factors including the application method, the detailed shape of the crop, and the initial adhesion amount [17,18,19,20].

**Figure 1:**
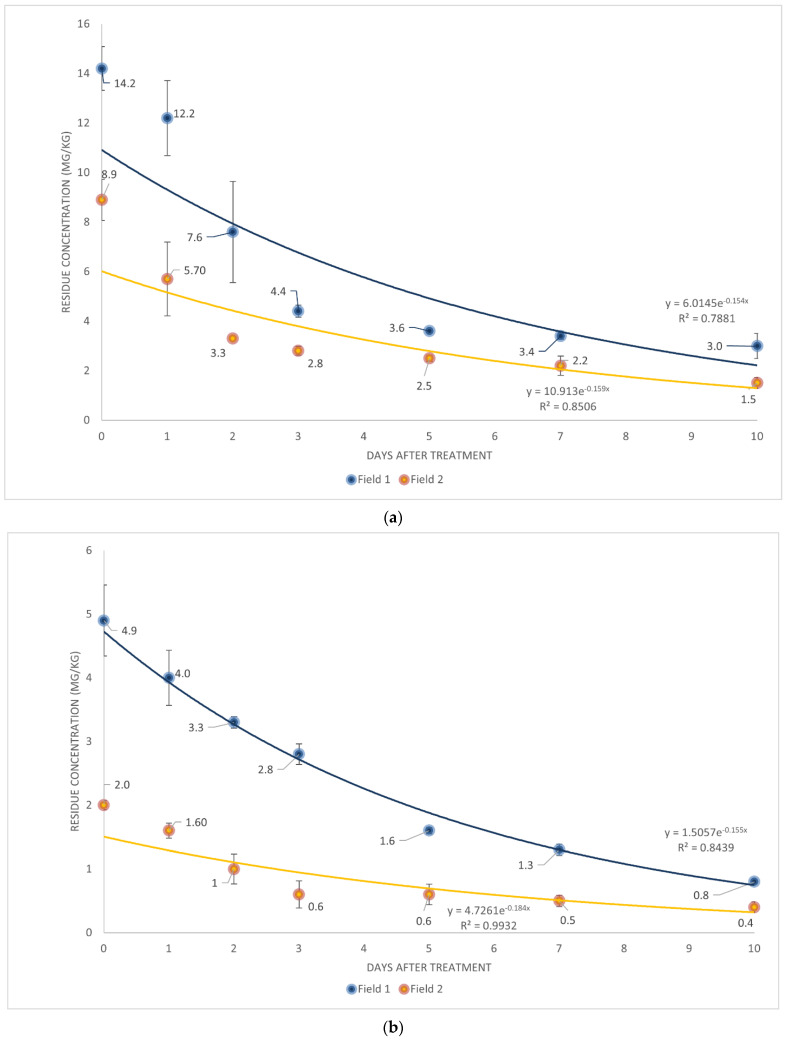
Dissipation kinetics of (**a**) spirodiclofen and (**b**) tebufenpyrad.

The regression analysis was utilized to determine the changes in the residue amount over time after treatment with spirodiclofen and tebufenpyrad during the cultivation period of *Aster scaber*. In the present study, during the cultivation period of *Aster scaber,* an exponential decrease in spirodiclofen and tebufenpyrad residue amount was observed. On the 10th day, the last day of harvesting, the initial residue amount of spirodiclofen was 21.1% for Field 1 and 16.9% for Field 2, whereas that of tebufenpyrad was 16.3% for Field 1 and 20.0% for Field 2 (Figure 1). The PHIs of spirodiclofen and tebufenpyrad for *Aster scaber* were seven days. Seven days after the harvest date, the residue amount of spirodiclofen was 3.4 and 2.2 mg/kg for Fields 1 and 2, respectively. In contrast, the residue amount of tebufenpyrad was 1.3 and 0.5 mg/kg for Fields 1 and 2, which were both <20.0 and 1.5 mg/kg (the MRL for each pesticide), respectively.

A regression formula for the daily residual amount in *Aster scaber* calculated through the coefficient of determination (r^2^) and simple regression analysis for both pesticides was >0.78, showing a high correlation (Figure 1). The biological half-lives of spirodiclofen in *Aster scaber* were calculated to be 4.4 and 4.5 days in Fields 1 and 2, respectively. In contrast, those of tebufenpyrad were 3.8 and 4.2 days, respectively, indicating no significant difference between the residue loss pattern and biological half-life of both pesticides. These results are consistent with those of a previous study on *Aster scaber* in which the half-lives ranged between 3.6 and 6.7 days [15,16], and are comparable to the half-lives of spirodiclofen in citrus, apple, peaches, and grapes (4.5–11.8 days) [21,22,23,24].

### 3.4. Calculation of the PHRL of Aster scaber

The PHRL refers to the residue amount at a specific point in time prior to harvesting such that the amount of pesticide residue should be below the MRL at the time of harvesting. PHRL is calculated by considering the lower limit of the 95% confidence level of the regression coefficient of the residue amount per day [16]. In the present study, the lower levels of the reduction constants for spirodiclofen were 0.0632 and 0.0723 for Fields 1 and 2, respectively. Whereas for tebufenpyrad, it was 0.1571 and 0.0868 for Fields 1 and 2, respectively. Table 4 displays the computed PHRLs based on these values. The PHRLs (10 days prior to harvest) of spirodiclofen for *Aster scaber* were 37.6 and 41.2 mg/kg for Fields 1 and 2, respectively. Meanwhile for tebufenpyrad, it was 7.2 and 3.6 mg/kg for Fields 1 and 2, respectively.

### 3.5. Risk Assessment

A risk assessment was conducted on the residues of spirodiclofen and tebufenpyrad in *Aster scaber*. Table 5 shows the results of the risk assessments of spirodiclofen and tebufenpyrad on days 0 and 7 after spraying according to the ingestion amount of *Aster scaber*. If the HQ is more than 100%, it indicates a high risk. In the case of spirodiclofen, the HQs on day 0 of the application were 178.9% (Field 1) and 112.1% (Field 2), whereas the HQs on day 7 of the application were 42.8% (Field 1) and 27.7% (Field 2). For tebufenpyrad, the HQs on day 0 of the application were 61.7% (Field 1) and 25.2% (Field 2), whereas the HQs on day 7 of the application were 16.4% (Field 1) and 6.3% (Field 2). On day 0, the hazard quotient (HQ) for spirodiclofen in both fields exceeded 100%, indicating a risk; however, on day 7 of the PHI period, the HQ decreased below 50%, indicating that it is not a high risk. In the case of tebufenpyrad, the HQ on days 0 and 7 of the application was less than 70%, making it less risky than spirodiclofen. However, the above HQ results were higher than the HQ range of 0.1%–3.9% found in a previous study on *Aster scaber* [25]. The sample collection period in their study was one year, obtained from large retailers and wholesale markets for agriculture products. Therefore, in agreement with previous studies, the present study suggests that consumption of *Aster scaber* in the production stage (0 and 7 days) has a lower risk.

## 4. Conclusions

The decreasing trend and residual characteristics of spirodiclofen and tebufenpyrad pesticides were identified in the *Aster scaber* production stage, and a risk assessment was conducted. While the initial residue amount differed in the residue reduction trend of the two fields, there was no difference in the half-lives because of similar cultivation conditions. All HQs were <100% in the risk assessment conducted using the residual amount on day 7 after pesticide application, corresponding to the PHI period of both pesticides, indicating that the risk is considered low. However, in the case of spirodiclofen, the HQs on days 0 and 7 after application were >100% and >25%, respectively, which could be considered a risk.

## Figures and Tables

**Table 1:** The formulation of spirodiclofen and tebufenpyrad investigated in the present study.

Pesticide	Formulation		Application				PHI ^c^ (Days)	MRL ^d^ (mg/kg)
	Type	AI ^a^	Dilution Rate	Spray No.	Interval (Days)	TSA ^b^		
Spirodiclofen	WP ^e^	36	4000	2	7	0.9	7	20.0
Tebufenpyrad	EC ^f^	10	2000	2	7	0.5	7	1.5

^a^ Active ingredient, %; ^b^ total sprayed amount of pesticides, g; ^c^ pre-harvest interval; ^d^ maximum residue limit; ^e^ wettable powder; ^f^ emulsifiable concentration.

**Table 2:** Recovery (RCV) and storage stability (STR) data for spirodiclofen and tebufenpyrad in *Aster scaber*.

Pesticides	Spiking Levels (mg/kg)		Recovery (%)				CV (%)	MLOQ (mg/kg)
			Replicates			(Mean ± SD)		
			1	2	3			
Spirodiclofen	RCV	0.1	101.4	96.1	103.1	100.2 ± 3.7	3.6	0.01
		1.0	98.0	115.9	98.4	104.1 ± 10.2	9.8	
	STR	1.0	101.8	103.6	100.0	101.8 ± 1.8	1.8	
Tebufenpyrad	RCV	0.1	82.0	98.1	87.1	89.1 ± 8.2	9.2	0.01
		1.0	106.0	102.0	101.8	103.3 ± 2.4	2.3	
	STR	1.0	101.4	109.8	101.3	104.2 ± 4.9	4.7	

SD: standard deviation; CV: coefficient of variation; and MLOQ: method limit of quantification.

**Table 3:** Normalized values (NVs) ^a^ of spirodiclofen and tebufenpyrad in *Aster scaber*.

Pesticides	Fields	Harvest Time (Days after Spraying the Pesticides)							
		0	1	2	3	5	7	10	Half- Lives
Spirodiclofen	1	15.8 ^a^	13.6	8.4	4.9	4.0	3.8	3.3	4.4
	2	9.9	6.3	3.7	3.1	2.8	2.4	1.7	4.5
Tebufenpyrad	1	9.8	8.0	6.6	5.6	3.2	2.6	1.6	3.8
	2	4.0	3.2	2.0	1.2	1.2	1.0	0.8	4.2

^a^ NVs are calculated as the residue of pesticides (mg/kg)/TSA (total sprayed amount; g).

**Table 4:** Recommended pre-harvest residue limits (PHRLs) of spirodiclofen and tebufenpyrad in *Aster scaber*.

Pesticide		Recommended PHRLs (mg/kg)				MRLs (mg/kg)
		10 Days Before Harvesting	7 Days Before Harvesting	5 Days Before Harvesting	3 Days Before Harvesting	
Spirodiclofen	Field 1	37.6	31.1	27.4	24.2	20.0
	Field 2	41.2	33.2	28.7	24.8	
Tebufenpyrad	Field 1	7.2	4.5	3.3	2.4	1.5
	Field 2	3.6	2.7	2.3	2.0	

**Table 5:** Risk assessment of spirodiclofen and tebufenpyrad in *Aster scaber*.

Pesticide			Residue Value (mg/kg)	ADI ^a^ (mg/kg bw/Day)	EDI ^b^ (mg/kg bw/Day)	HQ ^c^ (%)
Spirodiclofen	Day 0	Field 1	14.2	0.01	0.18	178.9
		Field 2	8.9	0.01	0.11	112.1
	Day 7 day	Field 1	3.4	0.01	0.004	42.8
		Field 2	2.2	0.01	0.003	27.7
Tebufenpyrad	Day 0	Field 1	4.9	0.01	0.006	61.7
		Field 2	2.0	0.01	0.003	25.2
	Day 7	Field 1	1.3	0.01	0.002	16.4
		Field 2	0.5	0.01	0.001	6.3

^a^ Acceptable daily intake; ^b^ estimated daily intake; ^c^ hazard quotient; HQ = EDI/ADI.

## Data Availability

Data will be made available on request.

## Appendix A

**Table A1:** Chemical Properties of Spirodiclofen and Tebufenpyrad [7].

Pesticide	Spirodiclofen	Tebufenpyrad
Chemical structure		
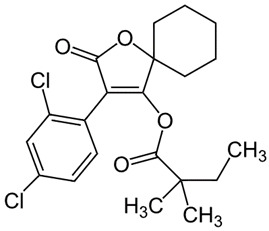
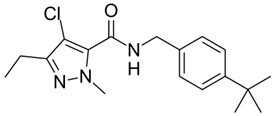
Vapor pressure	<3.0 × 10^−4^ mPa (20 °C)	<1.0 × 10^−2^ mPa (25 °C)
log K_ow_	5.1	4.9
Water solubility	0.19 mg/L (20 °C)	2.61 mg/L (25 °C)
Stability (DT_50_)	52.1 day (hydrolysis)	Stable to hydrolysis

**Table A2:** Analytical conditions of spirodiclofen and tebufenpyrad in *Aster scaber*.

Pesticides	Spirodiclofen			Tebufenpyrad		
Instrument	Shimadzu LC-MS 8045 (Shimadzu, Tokyo, Japan)					
Column	Kinetex C18 (100 × 2.1 mm, 2.6 μm, Phenomenex)					
Mobile phase	A: 0.1% formic acid in distilled water B: 0.1% formic acid in acetonitrile					
	Time (min)	A (%)	B (%)	Time (min)	A (%)	B (%)
	0.5	60	40	3	20	80
	2	5	95	3.5	10	90
	5	5	95	5.5	10	90
	6	60	40	6	20	80
	7	60	40	7	20	80
Flow rate	0.2 mL/min					
Injection volume	2 μL					
Retention time	4.19 min			1.47 min		
Detector	Triple-quadruple system					
Multiple reaction monitoring (MRM) parameters	Precursor ion > Product ion (collision energy voltage)			Precursor ion > Product ion (collision energy voltage)		
	Quantifier ion; 410.9 > 71.2 (−23.0)			Quantifier ion; 334.0 > 145.1 (−29.0)		
	Qualifier ion; 410.9 > 313.0 (−13.0)			Qualifier ion; 334.0 > 117.1 (−35.0)		
